# Glioblastoma induces CAF-like astrocyte activation via the AKT/mTOR–SERPINH1/COL5A1 axis

**DOI:** 10.17305/bb.2025.11898

**Published:** 2025-06-12

**Authors:** Jingxian Zhang, Yajia Chen, Hongwu Xu

**Affiliations:** 1 Departmant of Human Anatomy, Shantou University Medical College, Jinping District, Shantou, Guangdong, China; 2 Neurosurgery Department, The Tenth Affiliated Hospital, Southern Medical University, Dongguan, Guangdong Province, China

**Keywords:** Glioblastoma, cancer-associated fibroblasts, CAFs, astrocytes, SERPINH1, COL5A1, AKT/mTOR pathway

## Abstract

Glioblastoma multiforme (GBM), the most aggressive form of glioma, remains the most malignant tumor of the central nervous system. Despite a range of therapeutic strategies, the prognosis for GBM patients remains poor, underscoring the urgent need for novel treatments to inhibit GBM progression. The tumor microenvironment (TME) plays a critical role in tumor development, with cancer-associated fibroblasts (CAFs) acting as key components. However, the origin, composition, and spatial distribution of CAFs within the GBM microenvironment remain poorly understood. To address this gap, our research aims to investigate the etiology, cellular composition, and precise localization of CAFs in GBM, with the goal of elucidating their role in oncogenesis and tumor progression, thereby providing new avenues for therapeutic intervention. In this study, we developed a novel CAF-related prognostic model using data from the The Cancer Genome Atlas and Gene Expression Omnibus databases and identified *SERPINH1* and *COL5A1* as CAF-related genes in GBM. We established a GBM mouse model as well as a GBM cell and astrocyte co-culture system to examine the expression of *SERPINH1* and *COL5A1* in astrocytes under a simulated TME. Our findings revealed that these genes were more highly expressed in peritumoral tissue compared to normal brain tissue and showed strong co-localization with astrocytes. Furthermore, we found that normal astrocytes can be induced by GBM cells to activate the AKT/mTOR signaling pathway, migrate to the peritumoral region, and upregulate CAF-associated proteins (SERPINH1/COL5A1). These results suggest that astrocytes may serve as a potential source of CAF precursor cells within the GBM TME.

## Introduction

Glioblastoma multiforme (GBM), also known as glioblastoma or malignant glioma, accounts for approximately 57% of all gliomas and 48% of all primary malignant central nervous system (CNS) tumors [1]. Despite recent advances in treatment—including surgical resection, radiotherapy, systemic therapies (chemotherapy, targeted therapy), and supportive care—the overall prognosis remains poor, with low long-term survival rates. The median overall survival for GBM patients is approximately 12–18 months [2]. GBM primarily arises from glial cells, though it can also originate from neural stem cells [3]. Temozolomide (TMZ), a second-generation anti-cancer drug capable of crossing the blood–brain barrier (BBB), is currently the first-line chemotherapeutic agent for GBM. However, resistance to TMZ develops rapidly, and tumors frequently relapse after treatment [4, 5]. In response, recent research has shifted focus toward both direct GBM treatment and the tumor microenvironment (TME). The TME comprises tumor cells, surrounding immune cells, and associated stromal components, including the extracellular matrix (ECM), stromal cells (such as fibroblasts, immune, inflammatory, endothelial, and bone marrow-derived cells), cytokines, and chemokines [6–9]. It has been implicated in GBM resistance to targeted therapies [10], intra-tumor heterogeneity [11], and invasiveness [12]. Understanding the interactions between GBM and the TME could reveal new therapeutic targets. Cancer-associated fibroblasts (CAFs) are key components of the TME. They contribute to ECM deposition and remodeling, immunomodulation, angiogenesis, metabolic reprogramming, and overall TME regulation [13–16]. CAFs originate from diverse sources, including intrinsic fibroblasts, tumor epithelial and endothelial cells, and normal epithelial tissue [16]. They exhibit considerable heterogeneity and express a range of markers [16]. Increasing evidence supports the involvement of CAFs in tumor development, progression, immunosuppression, and resistance to therapy. Current investigations focus on identifying CAF subtypes and functions through marker analysis (e.g., FAP, α-SMA) and the detection of secreted factors such as IFN-γ and TGF-β, which vary with cancer stage and context [17, 18]. A recent study by Phillip et al. used single-cell sequencing and bioinformatics to identify a small population of CAFs within GBM. Despite their low abundance, these CAFs were significantly associated with tumor grade and patient prognosis [19]. Given their importance across cancer types, the origin, composition, and spatial distribution of CAFs within the GBM microenvironment remain areas of active investigation. Exploring the interaction between CAFs and GBM could facilitate the development of novel anti-GBM therapies. To identify CAF-related genes specifically expressed in GBM, we integrated data from The Cancer Genome Atlas (TCGA) and Gene Expression Omnibus (GEO)-derived GBM datasets to construct a novel prognostic model. Using this model, we identified two CAF hub genes—SERPINH1 and COL5A1—that are expressed in GBM. A CAF scoring correlation analysis confirmed their relevance as CAF markers in this context. We validated the expression of SERPINH1 and COL5A1 using a GBM mouse model. Protein levels of both genes were elevated in peritumor tissue compared to normal brain tissue. Co-localization assays conducted on mouse brain sections revealed that these genes were highly expressed in activated astrocytes surrounding the tumor. *In vitro* co-culture experiments further demonstrated that GBM cells can recruit and activate astrocytes at the tumor periphery, inducing overexpression of SERPINH1 and COL5A1. Bioinformatic analysis and western blotting suggested that this activation occurs via the AKT/mTOR signaling pathway.In conclusion, our findings indicate that GBM regulates surrounding astrocytes through the AKT/mTOR–SERPINH1/COL5A1 axis, promoting their activation and transformation into CAF-like cells. These CAF-like astrocytes may play roles in various GBM-associated pathological processes and represent potential targets for future therapies.

## Materials and methods

### Data acquisition

Clinical information, as well as transcriptional and mutation data for GBM, were obtained from TCGA (https://cancergenome.nih.gov/) and GEO (https://www.ncbi.nlm.nih.gov/geo/) databases. The TCGA dataset included 600 tumor samples with clinical characteristics such as age, sex, survival status, survival time, and tumor grade IV. Mutation data from TCGA were also downloaded for subsequent analysis.Expression data from glioma patients were retrieved from the GEO database, including the probe matrix (GSE43378) and platform (GPL570) files. These data included clinical information such as age, sex, survival status, and survival time. Data from TCGA and GEO were independently annotated and processed to differentiate mRNA expression profiles for further analyses. The steps used in this procedure are outlined in the following flowchart.

### Fibroblast and TIDE scoring

Tumor immune dysfunction and exclusion (TIDE) (http://tide.dfci.harvard.edu/login/), developed by Jiang et al. [20], is a computational framework used to predict tumor immune escape mechanisms and resistance to immunotherapy. In our study, we utilized this framework to obtain TIDE and fibroblast scoring files from TCGA and GEO datasets. Perl was used to generate the TIDE scoring file based on the average recurring mRNA expression. Normal samples were removed, and the mRNA expression values were log-transformed. Fibroblast scoring was conducted using the R packages EPIC, MCPcounter, and xCell [21]. Model construction and subsequent bioinformatics analyses were based on genes screened by EPIC. Although the xCell algorithm can classify cell types in the TME, including CAFs, we found inconsistencies during analysis. Specifically, after filtering the common gene set and conducting survival analysis, the risk stratification trends in the xCell results were reversed compared to expectations. As a result, we concluded that the gene set identified by xCell was not suitable for further analysis and excluded this algorithm from the study. Subsequently, the TME was analyzed using the R package ESTIMATE, which includes stromal cell scoring. Fibroblast and stromal cell scores were then combined and exported. Differential analysis was performed using the R package limma.

### CAF scoring survival analysis

The clinical file, generated from the TCGA and GEO databases, included patient ID, survival time, and survival status. We then merged the clinical, fibroblast-scoring, and TIDE-scoring data and iteratively analyzed them to determine the optimal cut-off value. This value was used to classify clinical samples into high- and low-risk groups. Kaplan–Meier (K–M) analysis, performed using the “survival” R package, was applied across all datasets to assess the feasibility of this classification and to evaluate overall survival. Survival curves were plotted using the “survivor” and “survminer” R packages [22].

### WGCNA screen for CAF hub genes

From the integrated TCGA and GEO dataset files, the top 5000 genes with large fluctuations in expression were selected as reference for EPIC scoring. We used “WGCNA” in the R package to analyze all values, remove free values, and obtain the best power value (value ═ 8) after conversion. Similar modules (number of genes ═ 30) were merged after clustering using a threshold of 0.2. Then, 1000 genes were randomly selected and used to draw a module gene heat map. Finally, a correlation heat map was constructed to illustrate the relationships between modules and CAF scoring. For the obtained modules and genes, a geneSigFilter value of 0.4 and a moduleSigFilter value of 0.8 were set, and the final hub genes of each module were output.

### Functional enrichment analysis of the hub genes

GO analysis included three components: biological processes (BPs), cellular components (CCs), and molecular functions (MFs). The functional annotations of genes in the CC, MF, and BP categories were obtained from the GO database [23]. Additionally, the Kyoto Encyclopedia of Genes and Genomes (KEGG) (https://www.kegg.jp/)—a widely used resource that integrates chemical, genomic, and functional information—was employed to identify relevant biological pathways [24]. Accordingly, we used both GO and KEGG analyses to investigate the signaling pathways and biological functions most significantly enriched among the intersecting hub genes, utilizing the “clusterProfiler” package in R.

### Construction of prognosis-related hub genes model

After combining survival and gene expression data from TCGA, survival analysis was conducted using the “survival” and “survminer” packages in R. A one-way significance filter was applied with a threshold of *P* ≤ 0.05 to identify prognosis-related genes by cycling through core genes. Forest plots were generated based on the expression of genes that showed significance in univariate analysis. Next, the TCGA dataset was used as the training set, and the GEO dataset served as the test set for constructing the prognostic model. A LASSO regression model was built using the “glmnet” package in R, and a cross-validation curve was plotted to determine the optimal lambda value corresponding to the lowest cross-validation error. The resulting model formula was derived from the product of gene expression levels and their respective coefficients. Based on the median risk score, clinical samples were stratified into high- and low-risk groups for further analysis. The risk score was calculated as follows: Risk score ═ (0.0210157891801135 × SERPINH1 expression level) + (0.110327492669992 × COL5A1 expression level).

### Survival analysis

To evaluate the model’s predictive capacity, K–M analysis was performed on the high- and low-risk groups using the survival package in R. A variable was considered an independent prognostic factor if the *P* value in both univariate and multivariate Cox regression analyses was less than 0.05. K–M analysis was also used to assess potential differences in progression-free survival (PFS) between the high- and low-risk groups.

### Immunotherapy analysis and tumor mutation burden (TMB)

Immunotherapy analysis was performed by inputting the TIDE file and using the “plyr” package in R [25]. A TIDE plot was generated, and ROC curves were plotted. AUC values were calculated using the “pROC” package in R. Tumor mutation analysis and correlation with CAF scores were then carried out using the “maftools” package in R.

### Drug sensitivity analysis

The Genomics of Drug Sensitivity in Cancer (GDSC) database (https://www.cancerrxgene.org/) is the largest public resource for tumor cell drug sensitivity and genomic data related to anti-tumor therapies [26]. Drug sensitivity for high- and low-risk groups was predicted by downloading expression and drug sensitivity data from the GDSC using the R packages limma and ggpubr. A filtering threshold of *P* < 0.05 was applied. Lower IC50 values indicate greater tumor sensitivity to a given drug.

### Cancer Cell Line Encyclopedia (CCLE), Human Protein Atlas (HPA), and Chinese Glioma Genome Atlas (CGGA) database validation

The CCLE is an open-access database that provides multi-omics data on thousands of cancer cell lines, including information on genetic mutations, RNA splicing, and protein modifications [27]. From the CCLE, we downloaded GBM data and searched for model gene expression data, which we then compared to fibroblast expression profiles. The HPA is an open-access resource that integrates proteomic, transcriptomic, and systems biology data to map tissues, cells, and organs. It includes data not only on tumors but also on normal tissues and provides survival curves for cancer patients. In this study, we used the HPA to validate the expression patterns of our model genes in both tumor and normal tissues. The CGGA is another open-access database that combines genomic technologies with bioinformatics to comprehensively characterize the glioma genome in the Chinese population. We used the CGGA to enhance the completeness of data for our model genes.

### Cell culture and co-culture

The following cultured cell lines were used in this study: the human normal astrocyte cell line SVGP12 (Hunan Fenghui Biotechnology Co., Ltd.); the human GBM cell lines U87 (GBM-like, Hunan Fenghui Biotechnology Co., Ltd.), T98G (Fuheng Co., Ltd.), LN229 (Se Ou Biology Co., Ltd.), and U343 (MeilunBio Co., Ltd.); and the mouse GBM cell line G422-GFP-LUC (Hunan Fenghui Biotechnology Co., Ltd.). Cells were cultured and expanded in either DMEM or minimum essential medium (MEM), supplemented with 100 U/mL penicillin/streptomycin and 10% fetal bovine serum (FBS). All cells were maintained at 37 ^∘^C in a humidified atmosphere containing 5% CO_2_. G422-GFP-LUC cells were selected using 1 µg/mL puromycin.Human cell lines were authenticated using short tandem repeat (STR) profiling, while species identification for the G422-GFP-LUC mouse cells was provided by the supplier (Hunan Fenghui Biotechnology Co., Ltd.). Co-culture experiments were conducted using cell-culture plates with 0.4 µm transwell inserts (LABSELECT, LOT: 14112; PET, 24 mm, 0.4 µm pore size). SVGP12 cells were seeded in the upper chambers, while U87, T98G, LN229, or U343 cells were seeded in the lower chambers. Both upper and lower chambers were seeded at a density of 1.5 × 10^5^ cells. After cell attachment, the upper chambers were inserted into the corresponding wells. Cells were harvested after 72 h of co-culture.

### Cell function assay

Scratch assays were performed using six-well plates with 0.4 µm transwell chambers (LABSELECT, LOT: 14112, PET, 24 mm, 0.4 µm). SVGP12 cells were co-cultured with tumor cells (1.5 × 10^5^) for 72 h. After co-culture, the chambers were rinsed three times with PBS pre-warmed to 37 ^∘^C. The chambers were then removed and placed on the inverted lids of the six-well plates. Scratch lines were made using a 200 µL pipette tip. After scratching, the chambers were returned to their corresponding wells. The chambers were rinsed three more times with pre-warmed PBS, then 2 mL of complete DMEM culture medium was added to each well for incubation. Wound healing was assessed at 0, 12, and 24 h. Migration assays were conducted using 12-well plates with 12 µm transwell chambers (JETBIOFIL, LOT: TCS100024, PC, 24 mm, 12 µm). SVGP12 cells (1 × 10^5^) were seeded in the upper chambers, and tumor cells were cultured in the lower chambers. After a 72-h co-culture, the membranes between the transwell chambers were removed, followed by crystal violet staining and microscopy.

### Establishment of the GBM mouse model

Ten-week-old adult female BALB/c mice (18–22 g) were purchased from Guangdong Sijia Jinda Biotechnology Co. All animal procedures were approved by the Laboratory Animal Ethics Committee of Shantou University Medical College (SUMCSY2024-002, Supplementary data). Due to the strict ethical scrutiny of our research organization and the limited number of available clinical cases, we did not have immediate access to human GBM tissue samples for our experiments. Although we initially considered using normal human brain samples as a control group, ethical constraints similarly prevented access to such tissue. Therefore, we employed a mouse model as a substitute. Mice were housed in the animal facility of Shantou University Medical College. A total of 35 mice were used in the animal experiments. Of these, 21 mice were used to establish the GBM model and the corresponding normal controls, while the remaining mice served as backups. Mice were anesthetized using tribromoethanol, and the fur on their heads was shaved. Each mouse’s head was immobilized using a rodent stereotaxic apparatus (RWD, 68025, China). A midline scalp incision was made, and a burr hole was drilled into the skull (2 mm to the right and 1 mm anterior to the bregma). G422-GFP-LUC cells (1×10^5^) suspended in 5 µL of saline were injected at a rate of 0.5 µL/min into the burr hole using a microsyringe inserted 2 mm below the skull surface. GBM mice were imaged preoperatively (1 day prior) and postoperatively (days 3, 7, and 14) using a small animal *in vivo* imaging system (IVIS Kinetic, USA), following intraperitoneal injection of fluorescein potassium salt (15 mg/ml PBS, ST196, Beyotime, China). Tumor tissues, peritumoral tissues, and contralateral normal tissues were dissected under a microscope once tumors had grown sufficiently. In this study, the peritumoral area of GBM is defined according to Marc Aubry and colleagues as the peripheral brain zone and the interface zone. During tissue sampling, regions within 2–3 mm of the tumor tissue, as determined by microscopic examination, were considered peritumoral tissue [28].

### Quantitative real-time PCR

The collected SVGP12 and tumor cells were lysed using Trizol for RNA extraction, followed by cDNA synthesis using the HiScript II First Strand cDNA Synthesis Kit (+gDNA Wiper) (Vazyme, China). RT-qPCR was performed on an ABI 7500 Real-Time PCR system using ChamQ Universal SYBR qPCR Master Mix (Vazyme). Results were considered reliable when Ct values ranged from 10 to 35. Expression changes of SERPINH1 and COL5A1 were measured, with GAPDH serving as the reference gene. Data analysis was conducted using the 2^−ΔΔCT^ method. The primers used in this assay are listed in Table S1.

### Western blotting

The collected cells and animal tissues were lysed on ice for 30 min using RIPA buffer (Beyotime, P0013C) supplemented with 1 mM protease and phosphatase inhibitors (Beyotime, P1045), followed by centrifugation at 16,900 × g for 40 min. The resulting supernatants were collected. Electrophoresis was carried out using 4%–20% SDS-PAGE gels, and proteins were transferred onto a PVDF membrane (Millipore, Tullagreen, Carrigtwohill, Ireland). The membrane was blocked with 5% bovine serum albumin (BSA) (ST023, Beyotime, China) in TBST for 2 h and then incubated overnight at 4 ^∘^C with the following primary antibodies: SERPINH1 (sc-5293, Santa Cruz, 1:1000), COL5A1 (sc-133162, Santa Cruz, 1:1000), GAPDH (GB15002, Servicebio, 1:2000), AKT (#4685, CST, 1:1000), P-AKT (#4060, CST, 1:1000), mTOR (ET1608-5, HUABIO, 1:1000), P-mTOR (HA600094, HUABIO, 1:1000), FAP (AF5344, Affinity, 1:800), and S100A4 (CY5799, Abways, 1:1000).The membrane was then incubated for 3 h at 4 ^∘^C with the following secondary antibodies: HRP-conjugated Goat Anti-Mouse IgG(H+L) (A0216, Beyotime, 1:1000) and HRP-conjugated Goat Anti-Rabbit IgG(H+L) (A0208, Beyotime, 1:1000). Protein bands were visualized using ultra-high sensitivity ECL (BL520B, Biosharp, Anhui, China) and imaged with the Mini Chemi610 Chemiluminescent Imaging and Analysis System (SINSAGE, Beijing, China). Images were captured using the system’s proprietary software (SageCaptur), and grayscale intensity was quantified using ImageJ. Full exposure membrane images are provided in S1_raw_images.

### Multicolor immunohistochemistry and hematoxylin and eosin (HE) staining

The mice were fully anesthetized using tribromoethanol and perfused with 4% paraformaldehyde. Brains were rapidly removed and post-fixed in 4% paraformaldehyde for 48 h at 4 ^∘^C (G1101, Servicebio). Whole brains were embedded in paraffin and sectioned at a thickness of 3 µm using a microtome (Leica RM2235, Germany). Sections were mounted on slides and stored at room temperature. Tissues were dewaxed with xylene (5 min, twice) and rehydrated through a graded alcohol series (75%–100%, 5 min, once). A four-color multiplex fluorescent immunohistochemical staining kit (AFIHC024, AiFang Biological, Hunan, China) was used for staining. Slides were then incubated overnight at 4 ^∘^C with the following primary antibodies: SERPINH1 (sc-5293, Santa Cruz, 1:100), COL5A1 (WLH4136, Wanleibio, 1:100), GFAP (HA600094, HUABIO, 1:500), MBP (WL03919, Wanleibio, 1:50), IBA1 (sc-32725, Santa Cruz, 1:100), FAP (AF5344, Affinity, 1:50), and S100A4 (CY5799, Abways, 1:100). A species-appropriate anti-mouse/rabbit secondary antibody (AFIHCC024, AiFang) was then applied. Imaging was performed using a confocal microscope (ZEISS LSM800, Germany). Whole-brain fluorescence scans were conducted by Servicebio (Wuhan, China). HE staining was performed using a Hematoxylin and Eosin Staining Kit (C0105S, Beyotime), and scanned with an iScan Coreo slide scanner (Roche Diagnostics).

### Ethical statement

All animal treatments and experiments in this study were approved by the Laboratory Animal Ethics Committee of Shantou University Medical College (Ethical review number: SUMCSY2024-002; see Supplementary data).

### Statistical analysis

All bioinformatics analysis and data visualization were performed using the R statistical programming language (version 4.2.3). A correlation matrix was constructed using Spearman’s test. Differences were considered statistically significant at a *P* value of less than 0.05. All experiments were independently repeated more than three times. GraphPad Prism 8 was used for statistical analysis, and ImageJ was used for image processing. For all experimental data, Student’s *t*-test was used for comparisons between two groups, while one-way analysis of variance (ANOVA) was employed for comparisons among multiple groups. Each experiment was repeated three times, and the results are representative of three independent replicates. A *P* value of less than 0.05 was considered statistically significant. In the statistical figures, *P* values are indicated with asterisks, where * represents *P* < 0.05, ** represents *P* < 0.01, *** represents *P* < 0.001, and **** represents *P* < 0.0001.

## Results

### Identification of 2 CAF-related hub genes with prognostic significance

**Figure 1.**
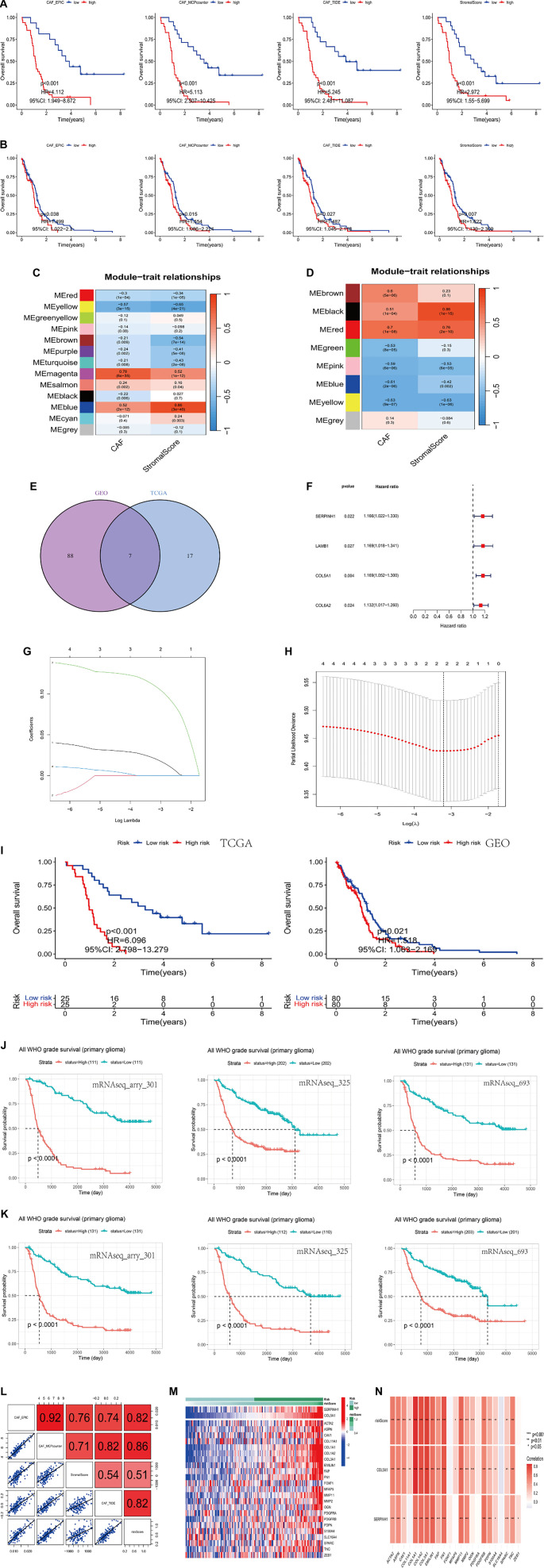
**Construction of CAF prognostic model, and gene screening and validation.** (A and B) Survival curves for TCGA (A) and GEO (B) data obtained using the four CAF-scoring algorithms; (C and D) Weighted correlation network analysis (WGCNA) of TCGA (C) and GEO data (D); Horizontal coordinates are the scored items, and vertical coordinates are the module names. Red color represents positive correlation, and blue represents negative correlation. The correlation coefficient is shown at the top of the module, and the *P* value used for correlation assessment is shown at the bottom. *P* < 0.05 is correlated with CAFs expression levels; (E) Intersection genes in TCGA and GEO datasets; (F) Univariate Cox regression analysis. Red indicates high risk; (G and H) Lasso Cox regression analysis (lasso Lambda and lasso Cvfit); (I) Kaplan–Meier curves for the survival analysis of the high- and low-risk groups in TCGA and GEO datasets, respectively; (J and K) Prognostic analyses of SERPINH1 (J) and COL5A1 (K) using mRNA data from the Chinese Glioma Genome Atlas (CGGA); (L) Correlation between CAF scores and patient risk scores. Correlation coefficient is shown at the top right. Scatter plot of correlations is shown in the lower left quadrant of the figure. The diagonal sequence of squares represents the type of scoring algorithm. The last column represents the correlation between the CAF scores and patient scores; (M) Heat map of two identified CAF-related genes and CAF genes reported in the literature; (N) Correlation analysis of CAF genes reported in the literature, the two identified CAF-related genes, and risk scores. CAF: Cancer-associated fibroblast; TCGA: The Cancer Genome Atlas; GEO: Gene Expression Omnibus.

Drawing upon established findings from prior research [29], we collected 606 GBM samples from TCGA and 50 GBM samples from the GEO. These tumor samples included clinical data such as age, sex, survival status, survival time, tumor grade (IV), and mutation profiles. The 606 TCGA samples were designated as the experimental group, while the 50 GEO samples served as a control group for validation. We evaluated both datasets using four algorithms—CAF-EPIC, CAF-MCPcounter, CAF-xCell, and stromal cell scoring (Tables S2 and S3). Based on their respective scores, samples were categorized into high- and low-scoring groups, followed by survival analysis. Except for results from the CAF-xCell algorithm, the low-scoring group consistently demonstrated better survival outcomes compared to the high-scoring group (Figure 1A and 1B), suggesting that CAFs may significantly influence GBM prognosis. Next, we applied WGCNA to identify gene modules related to CAFs in the high- and low-scoring groups and extracted the core genes (Figure 1C and 1D). By intersecting gene sets with the lowest *P* values, we identified seven core genes: SERPINH1, LAMC1, LAMB1, COL5A2, ADAM12, COL5A1, and COL6A2 (Figure 1E). Combining the seven core genes with survival data, we generated a forest plot (Figure 1F), identifying four high-risk prognostic genes: SERPINH1, LAMB1, COL5A1, and COL6A2. Using these, we constructed a prognostic model and refined it through LASSO regression and cross-validation, ultimately selecting SERPINH1 and COL5A1 as hub genes (Figure 1G and 1H). Survival analysis using these two genes showed that patients in the low-risk group had better outcomes than those in the high-risk group (Figure 1I), supporting the predictive validity of our model. To validate these findings externally, we used the CGGA-independent raw data analysis system [30]. Our results confirmed that SERPINH1 and COL5A1 were overexpressed in GBM (Figures S1 and S2), correlating with poorer overall survival (Figure 1J and 1K). Additional CGGA analyses are shown in Figures S3 and S4. Correlation analysis revealed a positive association between CAF scores and patient risk scores (Figure 1L), with the MCPcounter algorithm showing the highest correlation (*r* ═ 0.86), indicating the potential of CAF scores as markers of GBM progression. Furthermore, when comparing the identified hub genes to previously reported CAF-related genes, we found consistent expression patterns in high-risk groups and a strong positive correlation (Figure 1M and 1N), reinforcing the relevance of our identified genes. We also conducted immunotherapy profiling, tumor mutation analysis, and drug sensitivity screening based on the two hub genes. TIDE analysis showed that the high-risk group had higher TIDE scores, indicating greater immune evasion and potentially worse responses to immunotherapy (Figure S5). However, no significant difference was observed in TMB between high- and low-risk groups. Drug sensitivity analysis identified daporinad, staurosporine, and sabutoclaxas the top three candidate drugs (Figure S5). In summary, we identified two CAF-related hub genes—SERPINH1 and COL5A1—that are significantly associated with GBM prognosis and may serve as potential biomarkers or therapeutic targets.

### *SERPINH1* and *COL5A1* are highly expressed in the GBM and peritumor tissue

**Figure 2.**
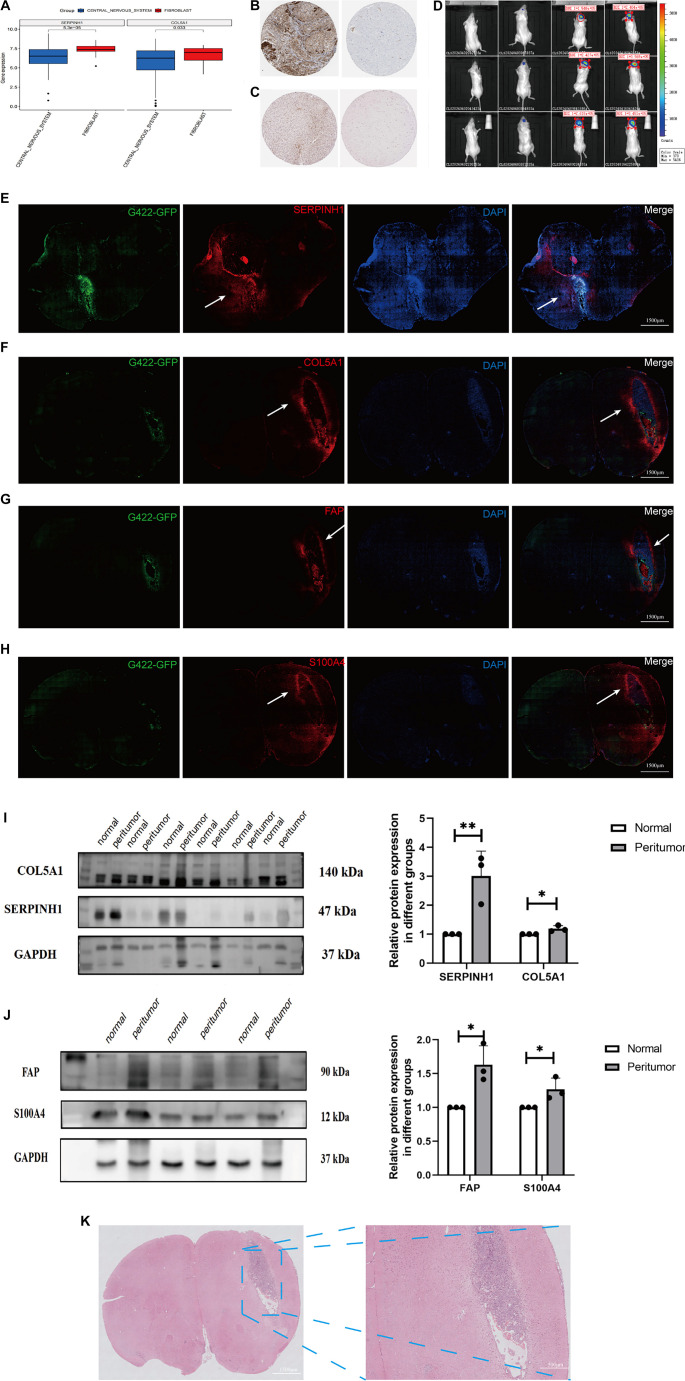
**Detection, localization, and expression of SERPINH1 and COL5A1**. (A) Expression of SERPINH1/COL5A1 in central nervous system (CNS) tumors and fibroblasts from the Cancer Cell Line Encyclopedia (CCLE) database; (B and C) Immunolabeling of SERPINH1 (B) and COL5A1 (C) in the GBM (left) and normal tissue (right) from the HPA database; (D) Tumor volumes were analyzed using bioluminescence imaging; (E–H) Multicolor IHC labeling of SERPINH1 (E), COL5A1 (F), FAP (G), and S100A4 (H) expression in the GBM mouse model. Green indicates G422-GFP expression. Red indicates the four CAF-associated proteins (SERPINH1, COL5A1, FAP, S100A4). Nuclei were stained using DAPI. Merge panel shows the combined image. White arrow indicates peritumor tissues with high expression of the indicated protein; (I–J) Western blotting analysis of the four CAF proteins in peritumor and normal tissues (FAP, 1.632 ± 0.279, *n* ═ 3; S100A4, 1.269 ± 0.165, *n* ═ 3; SERPINH1, 3.014 ±0.855, *n* ═ 3; COL5A1, 1.188 ±0.104, *n* ═ 3); (K) HE staining in the brain tissues of GBM mice. The experiment was repeated three times, and the results are representative of three independent experiments. The statistical results are shown in Figure S7. GBM: Glioblastoma multiforme; CAF: Cancer-associated fibroblast; HPA: Human Protein Atlas; HE: Hematoxylin and eosin.

CCLE and HPA databases were used to compare the expression of SERPINH1 and COL5A1 in normal and GBM tissues. Our results indicate that these two hub genes showed higher expression levels in fibroblasts than in GBM tissues based on CCLE data (*P* < 0.05, Figure 2A). In the HPA database, the protein expression of SERPINH1 and COL5A1 was higher in tumor tissues than in normal tissues (Figure 2B and 2C). Next, we evaluated the *in vivo* expression of these two genes. To simulate the complex brain microenvironment, we established an orthotopic G422-GFP-LUC GBM mouse model. Tumor implantation was confirmed via bioluminescence imaging (Figure 2D). Multi-color immunohistochemistry (mIHC) showed increased protein expression of SERPINH1 and COL5A1 in both tumor and peritumor tissues (Figure 2E and 2F), consistent with the results in Figure 2B and 2C. To validate the mIHC findings, we assessed SERPINH1 and COL5A1 protein expression in peritumor and contralateral normal brain tissue using western blotting. The results indicated higher expression levels in peritumor tissue compared to normal tissue (Figure 2I; *t*-test, *P* < 0.05, with statistical results shown on the right), aligning with the IHC findings. We then examined whether SERPINH1 and COL5A1 are specific markers of CAFs using two established CAF markers, FAP and S100A4. IHC and western blotting revealed that FAP and S100A4 were also more highly expressed in peritumor tissue than in normal tissue, mirroring the expression patterns of SERPINH1 and COL5A1 (Figure 2G, 2H, and 2J; *t*-test, *P* < 0.05; statistical data shown on the right). However, HE staining showed the presence of normal brain tissue cells in the peritumor region, with no specific cell staining or distinct pathological features (Figure 2K). These findings suggest that SERPINH1 and COL5A1 are indeed CAF-specific genes associated with GBM, and that their unique expression patterns may contribute to the complexity of GBM treatment. Additional statistical results are provided in Figure S7.

### GBM peritumor tissues recruit GFAP-positive astrocytes expressing CAF-related proteins

**Figure 3.**
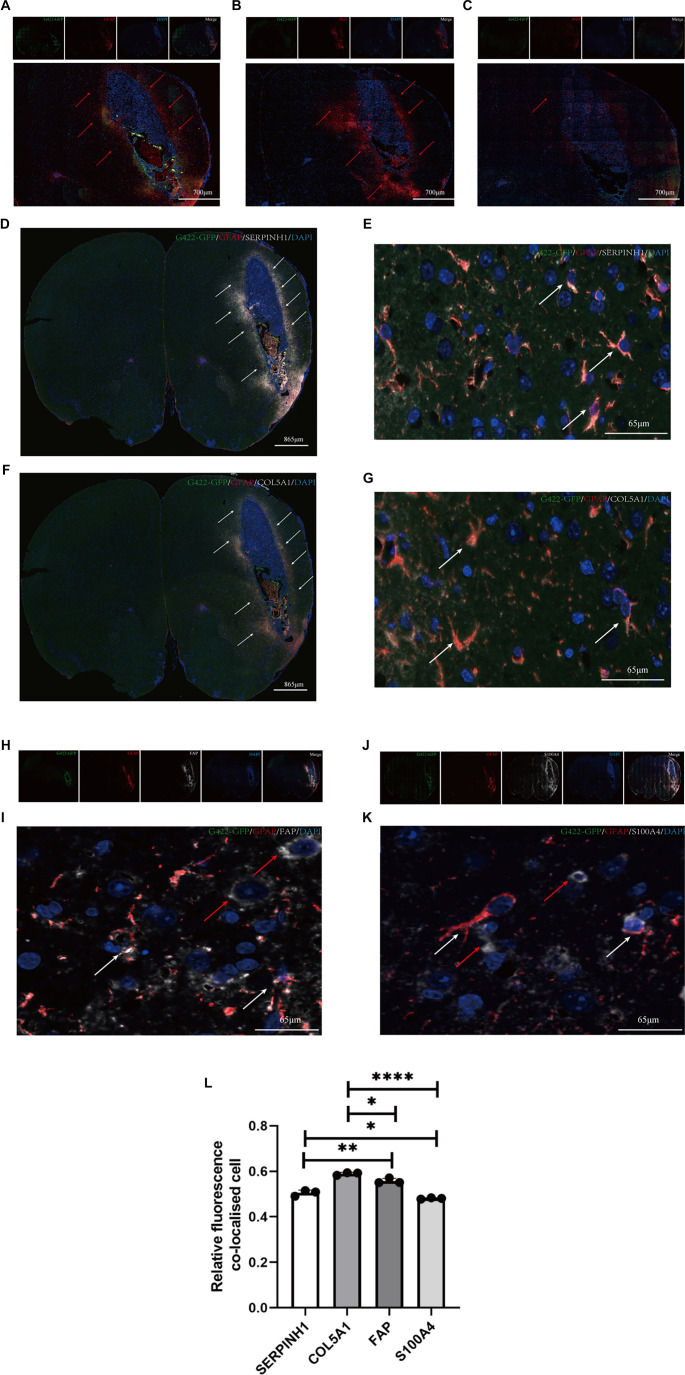
**Localization of SERPINH1 and COL5A1.** (A–C) Multicolor IHC staining of astrocytes (A, gfap), microglia (B, Iba1), and oligodendrocytes (C, MBP) in the GBM mouse model. Green indicates G422-GFP and Red indicates glial cells. Nuclei were stained using DAPI. The Merge panel shows the combined image. Red arrows indicate the localized recruitment of glial cells. The statistical results are shown in Figure S7. (D–G) Co-localization labeling of SERPINH1 (D, 10×; E, 63×) and COL5A1 (F, 10×; G, 63×) with astrocytes in the GBM mouse model. Green indicates G422-GFP, and Red indicates astrocytes. White indicates SERPINH1/COL5A1 expression. Nuclei were stained using DAPI. The Merge panel shows the combined image. White arrows indicate areas with high levels of co-localization and the cells that co-localized. (H–K) Co-localization labeling of FAP (H, 10×; I, 63×) and S100A4 (J, 10×; K, 63×) with astrocytes in the GBM mouse model. Green indicates G422-GFP, and Red indicates astrocytes. White indicates FAP/S100A4 expression. Nuclei were stained using DAPI. The Merge panel shows the combined image. White arrows indicate areas with high levels of co-localization and the cells that co-localized. Red arrows indicate FAP/S100A4-positive and GFAP-negative cells. (L) Statistical chart of tissue immunofluorescence localization (E, G, I, and K) results SERPINH1,0.557 ± 0.011, *n* ═ 3; COL5A1, 0.590 ± 0.006, *n* ═ 3; FAP, 0.505 ± 0.013, *n* ═ 3; S100A4, 0.481 ± 0.003, *n* ═ 3. The experiment was repeated three times, and the results are representative of three independent experiments. In the statistical figures, *P* values are indicated with asterisks, where *represents *P* < 0.05, ** represents *P* < 0.01, *** represents *P* < 0.001, and **** represents *P* < 0.0001. GBM: Glioblastoma multiforme.

To identify the CCs of peritumor tissue in the GBM mouse model, multicolor IHC was performed on whole-brain sections to label the three common types of glial cells: astrocytes, oligodendrocytes, and microglial cells. Interestingly, our results indicate that, compared with normal brain tissue, the peritumoral regions contain 30%–50% microglia, consistent with previous reports in the literature [31] (Figure 3A–3C, Figure S7; one-way ANOVA, *P* < 0.05). We also observed a substantial presence of astrocytes and a relatively low number of oligodendrocytes in these peritumoral regions (Figure 3A–3C, Figure S7; one-way ANOVA, *P* < 0.05). Based on these findings, we hypothesized that tumor presence may induce the migration and activation of astrocytes. To investigate this, we used a co-localization assay to evaluate the expression patterns of SERPINH1 and COL5A1 in astrocytes (Figure 3D–3G). Our results showed that both SERPINH1 and COL5A1 co-localized with GFAP-positive astrocytes, which were abundant in the peritumoral tissue. Furthermore, a co-localization assay assessing the expression of FAP and S100A4 in astrocytes revealed that these markers were expressed in both GFAP-positive and GFAP-negative astrocytes (Figure 3H–3K; one-way ANOVA, *P* < 0.05). These findings suggest that SERPINH1 and COL5A1 expression is more specific to GFAP-positive astrocytes. We therefore propose that GBM recruits a large number of GFAP-positive astrocytes to the peritumoral region during tumor progression and induces them to express CAF-related proteins. The corresponding statistical data are presented in Figure S7.

### GBM cells recruit astrocytes and induce them to express CAF-related proteins

**Figure 4.**
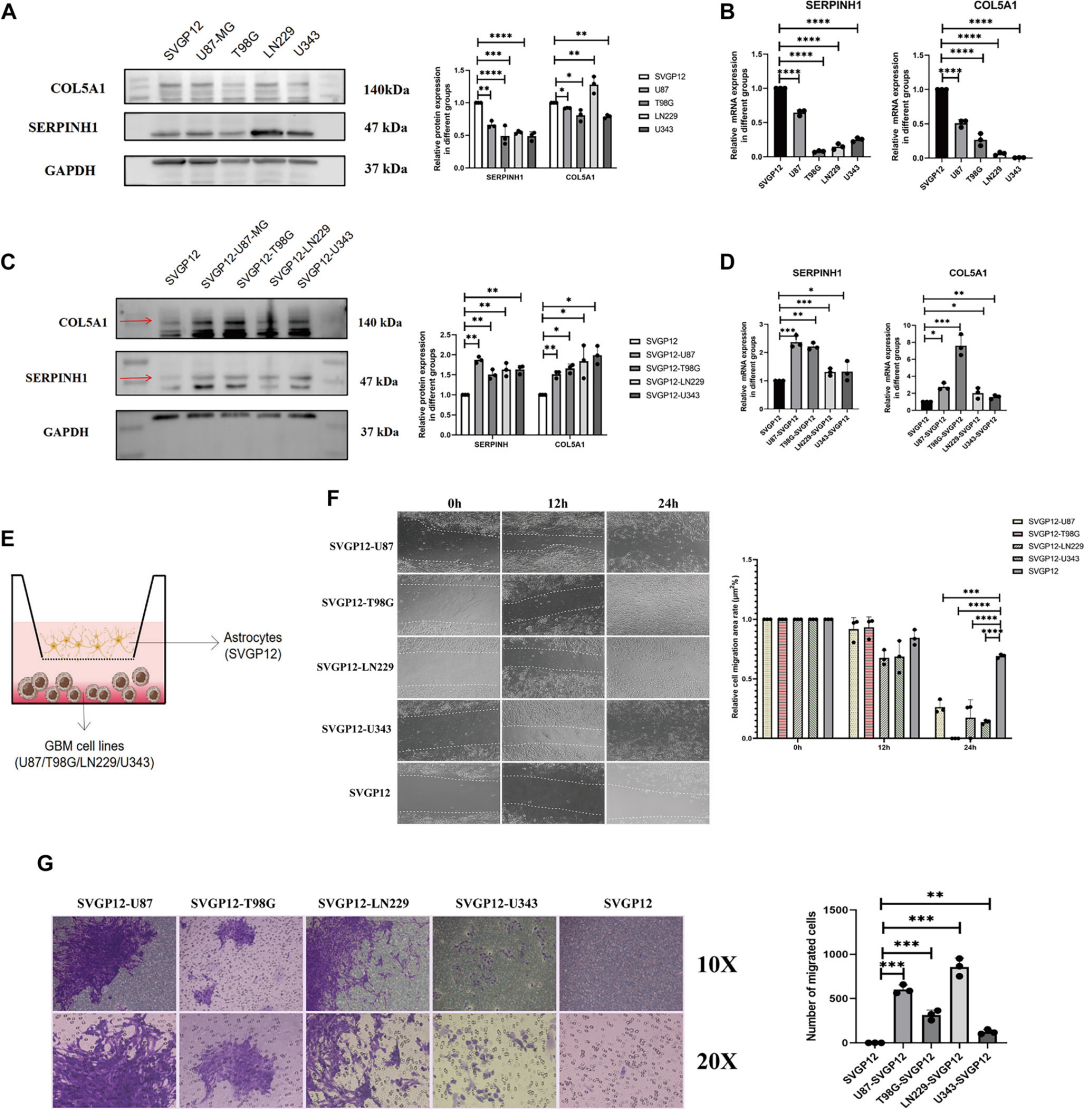
**The expression of SERPINH1 /COL5A1 and cell function assays in a GBM-astrocytes co-culture model.** (A and B) Western blotting (A) (SERPINH1: SVGP12, 1 ± 0, *n* ═ 3; U87, 0.664 ± 0.054, *n* ═ 3; T98G, 0.490 ± 0.146, *n* ═ 3; LN229, 0.547 ± 0.023, *n* ═ 3; U343, 0.487 ± 0.067, *n* ═ 3. COL5A1: SVGP12, 1 ± 0, *n* ═ 3; U87, 0.819 ± 0.169, *n* ═ 3; T98G, 0.809 ± 0.089, *n* ═ 3; LN229, 1.280 ± 0.130, *n* ═ 3; U343, 0.789 ± 0.026, *n* ═ 3) and RT-qPCR (B) (SERPINH1: SVGP12, 1 ± 0, *n* ═ 3; U87, 0.646 ± 0.041, *n* ═ 3; T98G, 0.077 ± 0.012, *n* ═ 3; LN229, 0.147 ± 0.042, *n* ═ 3; U343, 0.250 ± 0.029, *n* ═ 3. COL5A1: SVGP12, 1 ± 0, *n* ═ 3; U87, 0.509 ± 0.054, *n* ═ 3; T98G, 0.268 ± 0.089, *n* ═ 3; LN229, 0.065 ± 0.023, *n* ═ 3; U343, 0.007 ± 0.002, *n* ═ 3) in SVGP12 and GBM cell lines. (C and D) Western blotting (C) (SERPINH1: SVGP12, 1 ± 0, *n* ═ 3; SVGP12-U87, 1.871 ± 0.077, *n* ═ 3; SVGP12-T98G, 1.509 ± 0.117, *n* ═ 3; SVGP12-LN229, 1.628 ± 0.166, *n* ═ 3; SVGP12-U343, 1.635 ± 0.106, *n* ═ 3. COL5A1: SVGP12, 1 ± 0, *n* ═ 3; SVGP12-U87, 1.514 ± 0.088, *n* ═ 3; SVGP12-T98G, 1.659 ± 0.108, *n* ═ 3; SVGP12-LN229, 1.836 ± 0.410, *n* ═ 3; SVGP12-U343, 1.990 ± 0.228, *n* ═ 3) and RT-qPCR (D) (SERPINH1: SVGP12, 1 ± 0, *n* ═ 3; SVGP12-U87, 2.367 ± 0.217, *n* ═ 3; SVGP12-T98G, 2.211 ± 0.125, *n* ═ 3; SVGP12-LN229, 1.308 ± 0.128, *n* ═ 3; SVGP12-U343, 1.324 ± 0.348, *n* ═ 3. COL5A1: SVGP12, 1 ± 0, *n* ═ 3; SVGP12-U87, 2.759 ± 0.425, *n* ═ 3; SVGP12-T98G, 7.595 ± 1.252, *n* ═ 3; SVGP12-LN229, 2.027 ± 0.629, *n* ═ 3; SVGP12-U343, 1.561 ± 0.246, *n* ═ 3) in a co-culture model. (E) Schematic diagram of GBM-astrocytes in co-culture. (F) Wound healing assay in co-cultured SVGP12 (24 h: SVGP12, 0.692 ± 0.016, *n* ═ 3; U87, 0.264 ± 0.051, *n* ═ 3; T98G, 0 ± 0, *n* ═ 3; LN229, 0.174 ± 0.151, *n* ═ 3; U343, 0.137 ± 0.017, *n* ═ 3). (G) Migration assay in co-cultured SVGP12. (SVGP12, 1 ± 1, *n* ═ 3; U87, 603 ± 49.96, *n* ═ 3; T98G, 314 ± 55.05, *n* ═ 3; LN229, 856.7 ± 100.3, *n* ═ 3; U343,124.0 ± 26.21, *n* ═ 3). The statistical results are shown in Figure S7. The experiment was repeated three times, and the results are representative of three independent experiments. In the statistical figures, *P* values are indicated with asterisks, where *represents *P* < 0.05, **represents *P* < 0.01, ***represents *P* < 0.001, and ****represents *P* < 0.0001. GBM: Glioblastoma multiforme.

The mRNA and protein expression levels of SERPINH1 and COL5A1 in GBM cell lines and astrocytes (SVGP12), assessed using RT-qPCR and western blotting (Figure 4A and 4B; one-way ANOVA, *P* < 0.05; statistical results shown on the right), were inconsistent. While mRNA levels of both genes were higher in SVGP12 cells compared to GBM cell lines, SERPINH1 protein expression was significantly elevated in the LN229 GBM cell line relative to SVGP12 and other GBM lines—likely due to post-transcriptional regulation. These findings indicate that SERPINH1 and COL5A1 expression is not specifically elevated in GBM. To determine whether GBM cells could induce astrocytes to express CAF-related proteins, SVGP12 cells were co-cultured with human GBM cell lines using a transwell assay (Figure 4E). RT-qPCR and western blotting revealed that SERPINH1 and COL5A1 expression—both mRNA and protein—was elevated in co-cultured SVGP12 cells compared to untreated controls (Figure 4C and 4D; one-way ANOVA, *P* < 0.05; statistical results shown on the right). To test whether GBM cells recruit astrocytes, wound healing and migration assays were performed on co-cultured SVGP12 cells. The wound healing assay showed a significantly reduced scratch width in co-cultured groups (Figure 4F; one-way ANOVA, *P* < 0.05), and the migration assay demonstrated increased cell migration from the upper to the lower surface (Figure 4G; one-way ANOVA, *P* < 0.05). These results suggest that astrocytes in the co-culture gained enhanced migratory capacity.In summary, GBM cells appear to recruit astrocytes and induce CAF-related protein expression in them. Supporting statistical data are presented in Figure S7.

### The AKT/m-TOR pathway mediates the induction of astrocytes by GBM cells

**Figure 5.**
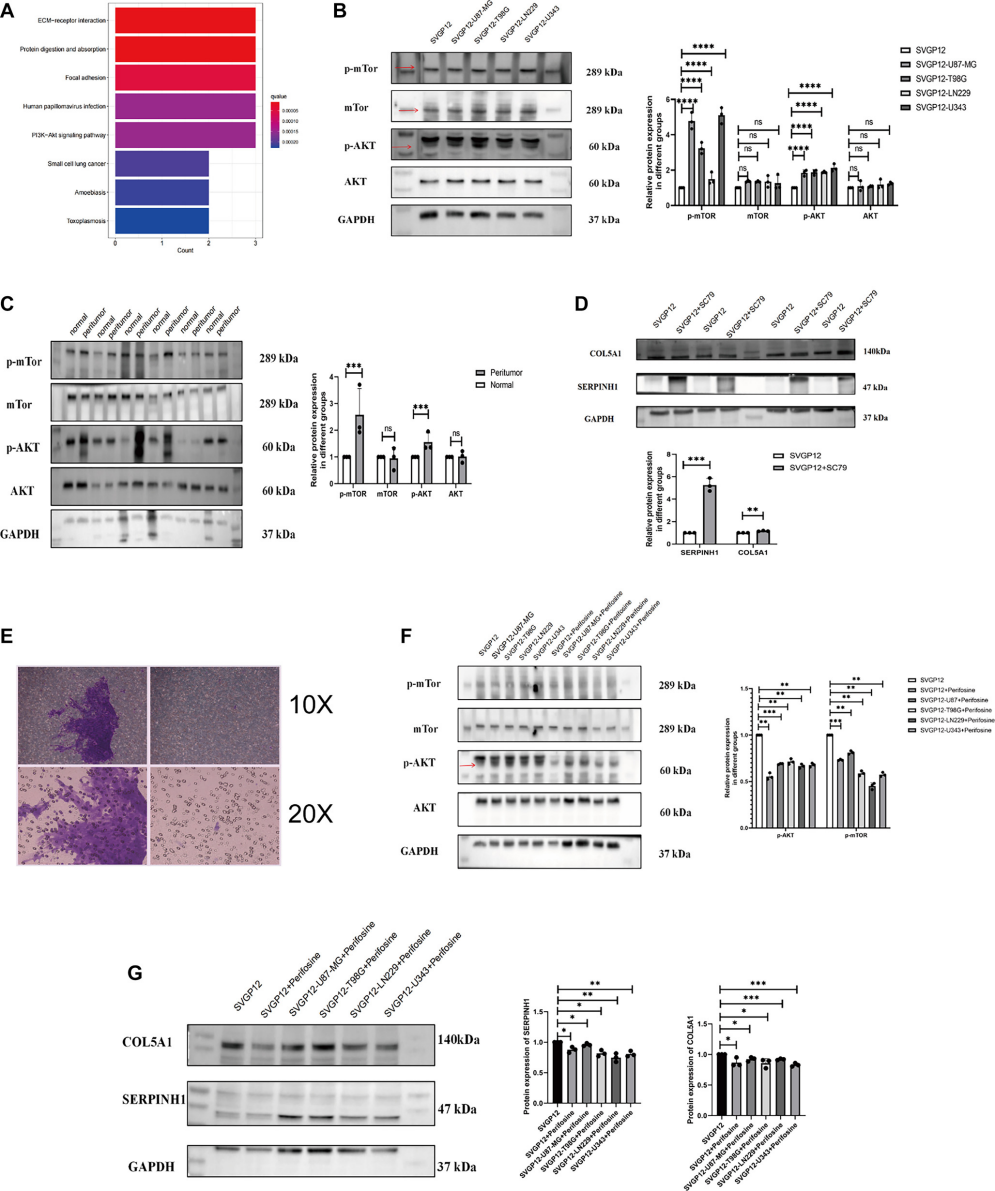
**The expression of SERPINH1/ COL5A1 and AKT/m-TOR pathway in a GBM and astrocytes co-culture model.** (A) KEGG pathway enrichment. (B) Western blot shows the expression of the AKT/m-TOR pathway in co-cultured SVGP12 cells (p-mTOR: SVGP12, 1 ± 0, *n* ═ 3; SVGP12-U87, 4.776 ± 0.467, *n* ═ 3; SVGP12-T98G, 3.234 ± 0.316, *n* ═ 3; SVGP12-LN229, 1.497 ± 0.349, *n* ═ 3; SVGP12-U343, 5.107 ± 0.456, *n* ═ 3. mTOR: SVGP12, 1 ± 0, *n* ═ 3; SVGP12-U87, 1.367 ± 0.054, *n* ═ 3; SVGP12-T98G, 1.349 ± 0.043, *n* ═ 3; SVGP12-LN229, 1.350 ± 0.330, *n* ═ 3; SVGP12-U343, 1.261 ± 0.438, *n* ═ 3. p-AKT: SVGP12, 1 ± 0, *n* ═ 3; SVGP12-U87, 1.847 ± 0.168, *n* ═ 3; SVGP12-T98G, 1.880 ± 0.152, *n* ═ 3; SVGP12-LN229, 1.877 ± 0.053, *n* ═ 3; SVGP12-U343, 2.127 ± 0.213, *n* ═ 3. AKT: SVGP12, 1 ± 0, *n* ═ 3; SVGP12-U87, 1.089 ± 0.287, *n* ═ 3; SVGP12-T98G, 1.075 ± 0.044, *n* ═ 3; SVGP12-LN229, 1.183 ± 0.269, *n* ═ 3; SVGP12-U343, 1.237 ± 0.077, *n* ═ 3). (C) Western blot shows AKT/m-TOR pathway expression in peritumor and normal tissues of GBM mice (p-mTOR: Normal, 1 ± 0, *n* ═ 3; Peritumor, 2.578 ± 0.981, *n* ═ 3. mTOR: Normal, 1 ± 0, *n* ═ 3; Peritumor,0.944 ± 0.413, *n* ═ 3. p-AKT: Normal, 1 ± 0, *n* ═ 3; Peritumor, 1.554 ± 0.349, *n* ═ 3. AKT: Normal, 1 ± 0, *n* ═ 3; Peritumor, 1.011 ± 0.218, *n* ═ 3). (D) Western blot shows SERPINH1 and COL5A1 expression in SVGP12 cells treated with the AKT agonist sc79 (SERPINH1: SVGP12, 1 ± 0, *n* ═ 3; SVGP12+SC79, 5.263 ± 0.584, *n* ═ 3. COL5A1: SVGP12, 1 ± 0, *n* ═ 3; SVGP12+SC79, 1.160 ± 0.033, *n* ═ 3). (E) Migration of SVGP12 cells treated with the AKT agonist sc79 (right) and of untreated SVGP12 cells (left). (F and G) Western blot shows the expression of the AKT-mTOR pathway (F) (p-mTOR: SVGP12, 1 ± 0, *n* ═ 3; SVGP12+Perifosine, 0.782 ± 0.007, *n* ═ 3; SVGP12-U87+Perifosine, 0.812 ± 0.018, *n* ═ 3; SVGP12-T98G+Perifosine, 0.588 ± 0.026, *n* ═ 3; SVGP12-LN229+Perifosine, 0.451 ± 0.035, *n* ═ 3; SVGP12-U343+Perifosine, 0.574 ± 0.023, *n* ═ 3. p-AKT: SVGP12, 1 ± 0, *n* ═ 3; SVGP12+Perifosine, 0.555 ± 0.036, *n* ═ 3; SVGP12-U87+Perifosine, 0.690 ± 0.006, *n* ═ 3; SVGP12-T98G+Perifosine, 0.714 ± 0.030, *n* ═ 3; SVGP12-LN229+Perifosine, 0.665 ± 0.023, *n* ═ 3; SVGP12-U343+Perifosine, 0.677 ± 0.021, *n* ═ 3) and that of SERPINH1 and COL5A1 (G) in co-cultured SVGP12 cells, and in co-cultured SVGP12 cells treated with the AKT inhibitor perifosine (SERPINH1: SVGP12, 1 ± 0, *n* ═ 3; SVGP12+Perifosine, 0.876 ± 0.048, *n* ═ 3; SVGP12-U87+Perifosine, 0.959 ± 0.023, *n* ═ 3; SVGP12-T98G+Perifosine, 0.817 ± 0.050, *n* ═ 3; SVGP12-LN229+Perifosine, 0.748 ± 0.070, *n* ═ 3; SVGP12-U343+Perifosine, 0.807 ± 0.046, *n* ═ 3.COL5A1:SVGP12, 1 ± 0, *n* ═ 3; SVGP12+Perifosine, 0.868 ± 0.072, *n* ═ 3; SVGP12-U87+Perifosine, 0.923 ± 0.034, *n* ═ 3; SVGP12-T98G+Perifosine, 0.859 ± 0.074, *n* ═ 3; SVGP12-LN229+Perifosine, 0.920 ± 0.011, *n* ═ 3; SVGP12-U343+Perifosine, 0.830 ± 0.028, *n* ═ 3). (H) Statistical graph of changes in SERPINH1 and COL5A1 protein expression with the addition of perifosine. The experiment was repeated three times, and the results are representative of three independent experiments. The statistical results are shown in Figure S7. In the statistical figures, *P* values are indicated with asterisks, where * represents *P* < 0.05, ** represents *P* < 0.01, ***represents *P* < 0.001, and **** represents *P* < 0.0001. GBM: Glioblastoma multiforme; KEGG: Kyoto Encyclopedia of Genes and Genomes.

Pertinent to previous research, CAFs exert tumor-promoting effects across various cancer types, with their activity linked to several signaling pathways, notably PI3K/AKT/mTOR, WNT, and MAPK [32–34]. To further investigate this phenomenon, KEGG analysis was performed on seven hub genes (SERPINH1, LAMC1, LAMB1, COL5A2, ADAM12, COL5A1, COL6A2). The results showed that these genes were enriched in the AKT signaling pathway (Figure 5A), which regulates tumor cell growth, survival, proliferation, and migration. Western blotting was used to detect the protein expression of SERPINH1, COL5A1, p-AKT, and its downstream effector, p-mTOR, in co-cultured SVGP12 cells. Compared to the control group, p-AKT and p-mTOR levels were elevated in the co-culture group, while total AKT and mTOR levels remained unchanged (Figure 5B, one-way ANOVA, *P* < 0.05; statistical results shown on the right). These findings indicate that GBM cells activate the AKT pathway in SVGP12 cells, resulting in increased expression of SERPINH1 and COL5A1 (Figure 4C and 4D, one-way ANOVA, *P* < 0.05; statistical results shown on the right). Western blotting of GBM mouse tissue revealed increased expression of p-AKT and p-mTOR in the peritumoral region, with no significant changes in total AKT or mTOR expression (Figure 5C, *t*-test, *P* < 0.05; statistical results shown on the right). To examine the regulatory relationship between the CAF-specific genes SERPINH1 and COL5A1 and the AKT pathway, SVGP12 cells were treated with the AKT agonist SC79, and co-cultured SVGP12 cells were treated with the AKT inhibitor perifosine. Activation of p-AKT led to increased expression of SERPINH1 and COL5A1 in SVGP12 cells (Figure 5D, *t*-test, *P* < 0.05; statistical results shown below the figure), along with enhanced cell migration (Figure 5E). Conversely, inhibition of p-AKT in co-cultured SVGP12 cells reduced the expression of SERPINH1 and COL5A1 (Figure 5F and 5G, one-way ANOVA, *P* < 0.05; statistical results shown on the right). In summary, our results demonstrate that GBM cells recruit astrocytes to the peritumoral region and induce them to express the CAF-related proteins SERPINH1 and COL5A1 via the AKT pathway. This suggests that astrocytes may serve as a potential source of CAF precursor cells in the TME of GBM. Statistical results are provided in Figure S7.

## Discussion

GBM is the most malignant type of glioma. Despite advancements in science and technology that have led to some progress in glioma treatment, there is currently no cure, and the prognosis for patients remains poor [35]. GBM is highly invasive—even after complete surgical resection and adjuvant chemotherapy—which poses a significant challenge in treatment. Tumor recurrence typically occurs in or within a few centimeters of the resection cavity [36, 37]. A major contributor to the invasiveness and intractability of GBM is its TME [38]. CAFs, critical components of the TME, play diverse roles [15]. However, unlike other tumor types, GBM is unique in that fibroblasts are largely absent from the brain, except for a small population in the brain vasculature [39]. Evidence suggests that CAFs in GBM do not arise from tumor-invaded peripheral tissues but instead originate from local sources such as bone marrow-derived precursor cells or mesenchymal stem cells [40–43]. Therefore, the presence of CAFs in GBM is plausible. While several studies have identified cells in GBM expressing CAF-associated markers, no gene expression profiles have conclusively verified their identity as CAFs, nor has their biological role in GBM been clearly established [44–46]. Although in situ injection is a well-established mouse model of GBM, this study represents a novel attempt to examine the distribution of CAFs throughout the entire brain. In our study, we integrated data from TCGA and GEO to construct a prognostic model based on four CAF-related genes. This model revealed that patient survival in GBM is associated with the CAF score. Through WGCNA, SERPINH1 and COL5A1 were identified as CAF-related hub genes predictive of patient prognosis. SERPINH1, a member of the serine protease inhibitor superfamily, encodes heat shock protein 47—a collagen-specific molecular chaperone. SERPINH1 is abnormally expressed in multiple cancers and is associated with tumor progression, making it a potential prognostic marker [47]. It has been linked to poor outcomes in glioblastoma, gastric cancer, and lung cancer [48–50]. Acting as an oncogene in glioma, SERPINH1 promotes tumor growth and invasion, while its inhibition suppresses cell proliferation, migration, and invasion, and induces apoptosis. *In vivo* knockout of SERPINH1 has been shown to significantly reduce tumor growth [51]. As a prognostic biomarker in glioma, SERPINH1 is involved in tumor progression through pathways such as JAK-STAT and modulates the immune microenvironment. High SERPINH1 expression is associated with immune evasion and poor immunotherapy response, highlighting its potential as a target for personalized treatment [52]. Its positive correlation with immune cells and checkpoint molecules suggests its oncogenic effects may be mediated through immune dysfunction, reinforcing its relevance to immunotherapy strategies. COL5A1, a fibril-forming collagen, is involved in ECM formation and is closely related to type XI collagen [53]. Type V and XI collagens may form tissue-specific combinations of collagen chains. COL5A1 has also been linked to hypoxia [54], and collagen deposition is commonly regarded as a pathological feature of TME [55]. Chemoresistance has been associated with increased tissue stiffness caused by specific collagen cross-linking [56]. COL5A1 is overexpressed in multiple cancers and is generally associated with poor prognosis [57, 58]. It is co-expressed with genes involved in major histocompatibility complex presentation, immune activation and suppression, chemokine signaling, mismatch repair, and immune checkpoint regulation [57]. However, the precise role of COL5A1 in cancer remains poorly understood, and further studies are needed to clarify its function in tumor biology.

To investigate the effects of SERPINH1 and COL5A1 on GBM, we established an orthotopic GBM mouse model to simulate the complex brain microenvironment and validate the expression of these two genes. Both SERPINH1 and COL5A1 showed increased expression in the peritumoral tissue and were highly co-localized in surrounding astrocytes. This observation is supported by our *in vitro* studies, which demonstrated that astrocytes were recruited to the peritumoral area by GBM. Furthermore, these GBM-recruited astrocytes expressed the CAF-associated proteins SERPINH1 and COL5A1 via activation of the AKT pathway and may exert CAF-like functions. Although several studies have examined SERPINH1 and COL5A1 in the context of GBM, to the best of our knowledge, this is the first study to identify these genes using CAF scores and to predict their biological roles. Our GO and KEGG enrichment analyses indicated that SERPINH1 and COL5A1 are primarily involved in glial cell formation, structural development, and ECM emodeling. In the absence of visible fibroblasts in the brain [59, 60], our findings suggest that astrocytes could serve as a potential origin for CAFs. We propose that changes in the expression of SERPINH1 and COL5A1 may induce astrocytes to adopt CAF-like characteristics, potentially promoting tumor invasiveness and metastasis. Under normal physiological conditions, astrocytes play diverse roles, including providing trophic and mechanical support to neurons, facilitating synaptogenesis, maintaining synaptic homeostasis, pruning synapses via phagocytosis, contributing to blood–brain barrier formation, and supporting other homeostatic functions [61]. In our study, we observed significant recruitment of astrocytes in peritumoral regions, which expressed CAF-related markers, SERPINH1 and COL5A1. We speculate that these recruited astrocytes may acquire CAF-associated functions, contributing to glioma recurrence and therapeutic resistance. Astrocytes can be activated into two distinct phenotypes: the neurotoxic/pro-inflammatory A1 type and the neuroprotective/anti-inflammatory A2 type [62]. A1 astrocytes release pro-inflammatory and neurotoxic cytokines, contributing to neuronal damage, whereas A2 astrocytes secrete anti-inflammatory cytokines and neurotrophic factors [63]. Due to current experimental limitations, we were unable to definitively characterize the astrocyte phenotypes in our study. However, recent studies suggest that A1-type astrocytes interact closely with glioma cells and influence both tumor cells and the surrounding microenvironment via direct contact or secretion of bioactive molecules [64]. Therefore, we hypothesize that the peritumoral astrocytes observed in our model may predominantly belong to the A1 subtype and play a role in glioma progression. We also employed the MCPcounter algorithm as a scoring method (Figure S6). Although multiple hub genes were identified, both EPIC and MCPcounter consistently highlighted SERPINH1 and COL5A1 as the most relevant. Consequently, these two genes were selected for further investigation. Model construction and subsequent bioinformatics analyses were performed using genes identified by EPIC. Initially, we applied three well-recognized CAF scoring algorithms, including the xCell algorithm, which classifies various cell types within the TME, including CAFs. However, during the analysis, we observed that the risk stratification trend produced by the xCell-derived gene set was reversed—high-risk and low-risk groups showed opposite patterns compared to other algorithms. Based on these inconsistencies, we concluded that the xCell-derived gene set was unsuitable for our study and excluded it from further analysis. In our study, although astrocytes may represent precursor cells of CAFs in GBM, their specific role in the GBM immune microenvironment remains unclear. Our bioinformatics analysis revealed that the high-risk group exhibited higher TIDE scores, and prior literature indicates that CAFs play an immunosuppressive role in tumor progression [65]. This suggests that CAF-like astrocytes may exert immunosuppressive effects—a hypothesis that warrants further experimental validation. TMB analysis showed that CAFs had no significant impact on GBM mutation status, consistent with the prognosis results obtained from the CGGA dataset. This consistency supports the reliability of our prognostic findings. For the overall prognostic analysis, data were sourced from Europe, America, and Japan. Notably, all cases were grade IV GBM; however, survival prognosis based on the expression of SERPINH1 and COL5A1 in CGGA data tended to align more closely with grade III GBM. This discrepancy may be related to patient ethnicity or geographic location. During our research, Western blot analysis of SERPINH1 and COL5A1 revealed multiple bands, potentially due to protein degradation, splice variants, or antibody cross-reactivity. We used the widely accepted AKT inhibitor Perifosine to investigate changes in SERPINH1 and COL5A1 expression. Results showed that inhibition of the AKT pathway reduced the expression of both proteins in astrocytes and impaired their migratory capacity. Specific bands for SERPINH1 and COL5A1 are indicated by red arrows in Figure 4C. In conclusion, we used CAF scores to analyze GBM data from multiple sources and identified two CAF-related genes—SERPINH1 and COL5A1—that influence prognosis. These findings may provide valuable insights for the development of future targeted therapies. We believe this study contributes to the understanding of the molecular therapeutic potential of CAFs in GBM. Furthermore, localizing CAF-like astrocytes via SERPINH1/COL5A1 expression may help better delineate tumor extent during GBM diagnosis and treatment. In addition, modulating SERPINH1/COL5A1 expression may enhance the efficacy of AKT-inhibiting drugs against GBM. Although our study advances understanding of GBM, several limitations remain. Determining whether SERPINH1 and COL5A1 directly affectGBM prognosis requires further experimental validation with more precise and diverse samples derived from human tissues rather than a single cell line. Moreover, while bioinformatics served as a useful predictive tool, the origin and development of CAFs in GBM must be confirmed through extensive *in vivo* and *in vitro* studies. The role of CAFs in GBM is still not fully understood. Some studies suggest that CAFs can either promote [66, 67] or inhibit [68, 69] tumor growth, possibly through different pathways. While our work clarified the impact of GBM on astrocytes, we did not investigate the reciprocal effect—how astrocytes may influence GBM after undergoing these changes—which represents an important direction for future research.

## Conclusion

In our study, two CAF-related genes, SERPINH1 and COL5A1, were identified in GBM datasets and may influence the prognosis of GBM patients. We found that these genes were highly expressed in peritumoral tissue. Additionally, GBM was shown to recruit astrocytes to the peritumoral region through activation of the AKT/mTOR pathway, subsequently inducing these astrocytes to express CAF-related proteins, including SERPINH1 and COL5A1. These findings suggest that astrocytes may serve as a potential source of CAF precursor cells within the GBM TME. As a result, the distribution of CAF-like astrocytes—marked by SERPINH1 and COL5A1—could help more precisely define tumor boundaries during diagnosis and treatment. Furthermore, modulating SERPINH1/COL5A1 expression may enhance the efficacy of AKT-inhibiting therapies for GBM.

## Supplemental data

Supplemental data are available at the following link: https://www.bjbms.org/ojs/index.php/bjbms/article/view/11898/3966.

## Data Availability

We have uploaded the code and data from the manuscript to a database. The link and schematic representation are in Supplemental data.
