# Correction: Glioblastoma induces CAF-like astrocyte activation via the AKT/mTOR-SERPINH1/COL5A1 axis

**DOI:** 10.17305/bb.2025.13134

**Published:** 2025-08-16



Correction to: *Glioblastoma induces CAF-like astrocyte activation via the AKT/mTOR-SERPINH1/COL5A1 axis* [1].

This corrigendum corrects the authors' affiliations to:

Jingxian Zhang^1,2#^, Yajia Chen^1,2#^, and Hongwu Xu^1,2^*

^1^Department of Neurosurgery, Tenth Affiliated Hospital, Southern Medical University, Dongguan, Guangdong, China.

^2^Department of Human Anatomy, Shantou University Medical College, Jinping District, Shantou, Guangdong, China.

The authors sincerely apologize for this error and confirm that this correction does not affect the scientific content or conclusions of the article.
